# Thermoelectric performance of *n*-type Mg_2_Ge

**DOI:** 10.1038/s41598-017-04348-7

**Published:** 2017-06-21

**Authors:** Rafael Santos, Mitchell Nancarrow, Shi Xue Dou, Sima Aminorroaya Yamini

**Affiliations:** 1 Australian Institute for Innovative Materials (AIIM), Innovation Campus, University of Wollongong, Wollongong, NSW 2500 Australia; 2 Electron Microscopy Centre (EMC), Innovation Campus, University of Wollongong, Wollongong, NSW 2500 Australia

## Abstract

Magnesium-based thermoelectric materials (Mg_2_X, X = Si, Ge, Sn) have received considerable attention due to their availability, low toxicity, and reasonably good thermoelectric performance. The synthesis of these materials with high purity is challenging, however, due to the reactive nature and high vapour pressure of magnesium. In the current study, high purity single phase *n*-type Mg_2_Ge has been fabricated through a one-step reaction of MgH_2_ and elemental Ge, using spark plasma sintering (SPS) to reduce the formation of magnesium oxides due to the liberation of hydrogen. We have found that Bi has a very limited solubility in Mg_2_Ge and results in the precipitation of Mg_2_Bi_3_. Bismuth doping increases the electrical conductivity of Mg_2_Ge up to its solubility limit, beyond which the variation is minimal. The main improvement in the thermoelectric performance is originated from the significant phonon scattering achieved by the Mg_2_Bi_3_ precipitates located mainly at grain boundaries. This reduces the lattice thermal conductivity by ~50% and increases the maximum *z*T for *n*-type Mg_2_Ge to 0.32, compared to previously reported maximum value of 0.2 for Sb-doped Mg_2_Ge.

## Introduction

Over the last two decades, the search for high performance thermoelectric (TE) materials has been renewed due to increased awareness of energy losses, particularly as waste heat, and their negative contribution to general energy efficiency^1, 2^. Solid state thermoelectric generators have recently been adopted for large-scale applications such as the automotive^3^ and metal processing industries^4^. Nevertheless, the main hindrances to the worldwide implementation of TE generators are still their high cost and low conversion efficiencies. The efficiency of thermoelectric materials is defined by the figure of merit, *z*T, where $z{\rm{T}}={{S}^{2}\sigma T/\kappa }_{tot}$, *S* is the Seebeck coefficient, *σ* the electrical conductivity, *T* the absolute temperature, and *κ*_tot_ the total thermal conductivity. Improving *z*T while reducing the cost is currently the main aim of the research on thermoelectric materials and devices. Higher *z*T can be achieved by improving the power factor, $PF={S}^{2}\sigma$, and by reducing the total thermal conductivity, ${\kappa }_{tot}={\kappa }_{lat}+{\kappa }_{el}$ where *κ*_lat_ and *κ*_el_ are the lattice and electrical components of the thermal conductivity, respectively.

Mg_2_X compounds (X = Si, Ge, Sn) and their alloys are promising thermoelectric materials due to their low cost, low toxicity, and good *n*-type thermoelectric performance^5, 6^. These semiconductor materials are face-centred cubic CaF_2_ (Fm3m space group) with similar electronic band structures^7^ characterized by a split conduction band^8^. Amongst the binary compounds, *n*-type Mg_2_Si^9, 10^ and Mg_2_Sn^11^ exhibit inherently higher thermoelectric performance than Mg_2_Ge^12^. Following the high *z*T of 1.1 reported for the pseudo-binary Mg_2_Si-Mg_2_Sn system^8^, originating from mass-difference phonon scattering and conduction band convergence^8^, considerable efforts have been devoted to fabricating and characterising complex ternary^2, 13, 14^ and quaternary^2, 15, 16^ compounds. Whereas, binary Mg_2_Ge has received very limited attention, with only two experimental reports on *n*-type Sb-doped samples^12, 17^ and a single report on *p*-type Ag-doped Mg_2_Ge thin films^18^.

Here, we report the fabrication of Bi-doped Mg_2_Ge samples (Mg_2_Ge_1−*x*_Bi_*x*_, *x* = 0, 0.005, 0.010, 0.020, 0.030) as an alternative to Sb-doped Mg_2_Ge, via one-step spark plasma sintering of elemental Ge and Bi with MgH_2_. This method was previously used to avoid the long, high temperature traditional methods in the synthesis of Mg_2_Si^19^, with the objective of reducing the volatilization and oxidation of Mg. The electronic transport properties are compared with those of an Sb-doped sample and previous reports on Sb-doped Mg_2_Ge samples fabricated by melting techniques followed by hot-pressing^12, 17^. A maximum *z*T of 0.32 at 750 K for Mg_2_Ge_0.97_Bi_0.03_ was obtained, which was higher than the previously reported *z*T value of 0.2 for Sb-doped Mg_2_Ge^12^. This was achieved due to the lower thermal conductivity of this compound, originating from the bismuth-rich precipitates formed at the grain boundaries and embedded within the matrix. These results, however, suggest that there is very low solubility of Bi in Mg_2_Ge which limits its utility as an effective dopant.

## Methods

Magnesium hydride (Sigma-Aldrich, hydrogen storage grade), germanium (Alfa Aesar, 99.999%) and bismuth (Alfa Aesar, 99.999%) were mixed to obtain Mg_2_Ge_1−*x*_Bi_*x*_ (*x* = 0, 0.005, 0.010, 0.020, 0.030) and Mg_2_Ge_0.98_Sb_0.02_, with extra 10 at.% of MgH_2_ added in order to compensate for the Mg loss during synthesis. The powder mixture was ball-milled in argon filled tungsten carbide vials with a ball-to-powder mass ratio of 33:1 using a Fritch Pulverisette 7 premium line planetary ball mill. The powders were then loaded into a graphite die with a 12 mm inner diameter and sintered into discs approximately 2 mm thick using spark plasma sintering (SPS) in vacuum. The SPS procedure consisted of heating the powder to 623 K in 15 min at 50 MPa and maintaining that temperature for 20 minutes to ensure full decomposition of the MgH_2_. The temperature was then increased to 823 K in 5 minutes, followed by sintering for 30 minutes. All sintered samples were annealed in vacuum-sealed quartz tubes for 72 h at 723 K.

The crystal structure of samples was analysed with a GBC Scientific X-ray diffractometer (XRD) with Cu Kα radiation (*λ* = 1.544 Å, 40 kV, 25 mA). The lattice parameters of Mg_2_Ge_1−*x*_Bi_*x*_ were obtained by Rietveld refinement of the XRD patterns. The microstructure and phase composition analysis were performed by scanning electron microscopy (SEM), with a JEOL 7001F SEM equipped with energy dispersive spectroscopy (EDS) at 15 kV and Oxford Instruments X-Max^n^ 80 mm^2^ SSD detector and processed using the Aztec analytical software suite. The total thermal conductivity (*κ*_tot_) was calculated from ${\kappa }_{tot}=d\cdot D\cdot {C}_{p}$. The density (*d*) was calculated using the measured weight and dimensions, and the heat capacity (*C*_*p*_) was obtained from the literature^20–22^. The laser flash method (LFA) was used to measure the thermal diffusivity (*D*) using a Linseis LFA-1000, along the thickness of the disc-shaped samples. These were cut into parallelepiped-shaped samples where the Seebeck coefficient (*S*) and the electrical conductivity (*σ*) were measured with a Linseis LSR-3 Seebeck coefficient and electric resistivity measurement equipment using the slope method in quasi-steady-state mode with temperature differences of 1 to 10 K between the probes, perpendicular to the sintering direction. Samples with low electrical conductivity were measured in an ad-hoc apparatus using the Van der Pauw technique for determining their electrical conductivity and Seebeck coefficient. Hall Effect measurements of samples *x* = 0.010, 0.020 and 0.030 of Mg_2_Ge_1−*x*_Bi_*x*_ were performed at room temperature using a physical property measurement system (PPMS) from Quantum Design.

## Results and Discussion

**Figure 1:**
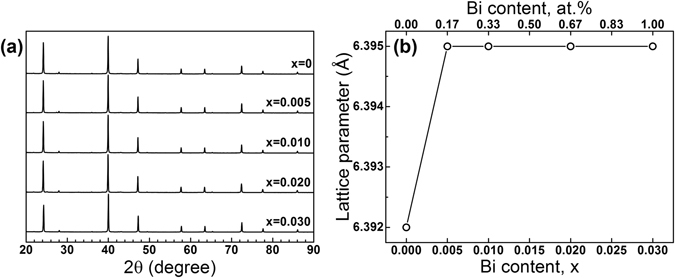
(**a**) XRD patterns and (**b**) lattice parameters of Mg_2_Ge_1−x_Bi_x_ samples obtained by Rietveld refinement. Note that the lattice parameters do not change upon greater Bi addition beyond 0.17 at.%.

Powder X-ray diffraction patterns of the Mg_2_Ge_1−*x*_Bi_*x*_ (*x* = 0, 0.005, 0.010, 0.020, 0.030) samples sintered by SPS show all samples were single-phase with the major reflections corresponding to the cubic CaF_2_ structure (space group Fm-3m) of Mg_2_Ge (Fig. 1(a)). A small peak corresponding to MgO (2θ = 42.97°) was detected in all samples while Mg_3_Bi_2_ was only detected in the sample with *x* = 0.020 at 2θ ≈ 25°. Although the formation of Mg_3_Bi_2_ was previously reported in Bi-doped Mg_2_Si_1−*x*_Ge_*x*_^23^ and Bi-doped Mg_2_Sn^11^, the solubility limit of Bi in the Mg-based group IV intermetallics is still unknown. The introduction of Bi significantly increased the lattice parameter up to *x* = 0.005 (0.17 at.%) (as shown in Fig. 1(b)), after which it remains constant, indicating that the Bi solubility limit has been reached in Mg_2_Ge in all samples.

**Figure 2:**
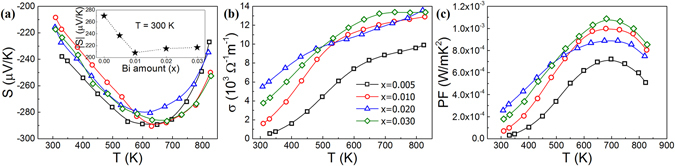
Temperature dependence of (**a**) the Seebeck coefficient, inset: room temperature Seebeck coefficient as a function of the added Bi; (**b**) the electrical conductivity, and (**c**) the power factor of the Mg_2_Ge_1−*x*_Bi_*x*_ (*x* = 0.005, 0.010, 0.020, 0.030) samples.

Temperature dependence of the Seebeck coefficients up to 825 K for all doped samples show that the absolute Seebeck coefficient increases with temperature up to approximately 630 K. After this, it decreases due to the bipolar effect, where the minority charge carriers contribute to the conduction due to thermal excitation, enough to bridge the band gap (Fig. 2(a)). The observation of this effect was likely due to a low charge carrier concentration and the narrow band-gap at these temperatures. The energy band gap, *E*_g_, of Mg_2_Ge at 0 K was reported to be 0.74 eV and to decrease rapidly at a rate of $-8\times {10}^{-4}$ eV with temperature^24^. This results in an energy gap in Mg_2_Ge equal to 0.26 eV at 630 K. Estimating the band-gap energy using ${E}_{g}=2e|{S}_{\max }|{T}_{\max }$ ^25^, where *e* is the elementary charge, *S*_*max*_ the maximum Seebeck coefficient, and *T*_*max*_ the temperature at which *S*_*max*_ is achieved, results in a band gap of $0.37\pm 0.07$ eV at 650 K for the sample with a Bi doping concentration of *x* = 0.005. Regardless of the precision of these estimates, both methods indicate a narrow energy gap (*E*_g_). Hall Effect measurements at room temperature of samples *x* = 0.010, 0.020 and 0.030 (Mg_2_Ge_1−*x*_Bi_*x*_) show the charge carrier concentration of $4.6\times {10}^{18}$, $5.7\times {10}^{18}$ and $7.7\times {10}^{18}$ cm^−3^, respectively (Table 1). The narrow energy gap (*E*_*g*_ *)* along with the low charge carrier concentration of these samples leads to the early onset of the bipolar effect. The energy gap enables the Seebeck coefficient to increase in absolute value up to the temperature where the energy difference is small enough to be crossed by thermally excited minority charge carriers. The room temperature Seebeck coefficient decreases with added Bi up to *x* = 0.010, after which it remains roughly constant (Fig. 2(a) inset). The temperature dependence of the electrical conductivity and power factor of the doped Mg_2_Ge samples are shown in Fig. 2(b) and (c), respectively. All the samples show an increase in the electrical conductivity with temperature, which is a typical behavior of non-degenerate semiconductors^26^. The plateau region of the electrical conductivity at temperatures above 600 K is another indication of the bipolar effect. At high temperature, Bi doping increases the electrical conductivity of the samples up to *x* = 0.010, while further increase of the dopant results in no significant difference. At room temperature, sample *x* = 0.020 exhibits higher electrical conductivity, despite sample *x* = 0.030 having a higher charge carrier density, due its higher carrier mobility (Table 1). The electrical conductivity of the undoped sample (*x* = 0) was found to be $\sigma =50.13$ Ω^−1^m^−1^, several orders of magnitude lower than for the doped samples and is, therefore, not shown in the relevant figures.

**Table 1:** Room temperature electron concentration, *n*_*H*_, and carrier mobility, *µ*_*H*_, of Mg_2_Ge_1–*x*_Bi_*x*_.

*x*	*n*_*H*_ (10^18^ cm^−3^)	*µ*_*H*_ (cm^2^/Vs)
0.010	4.6	21.93
0.020	5.7	60.40
0.030	7.7	30.61

**Figure 3:**
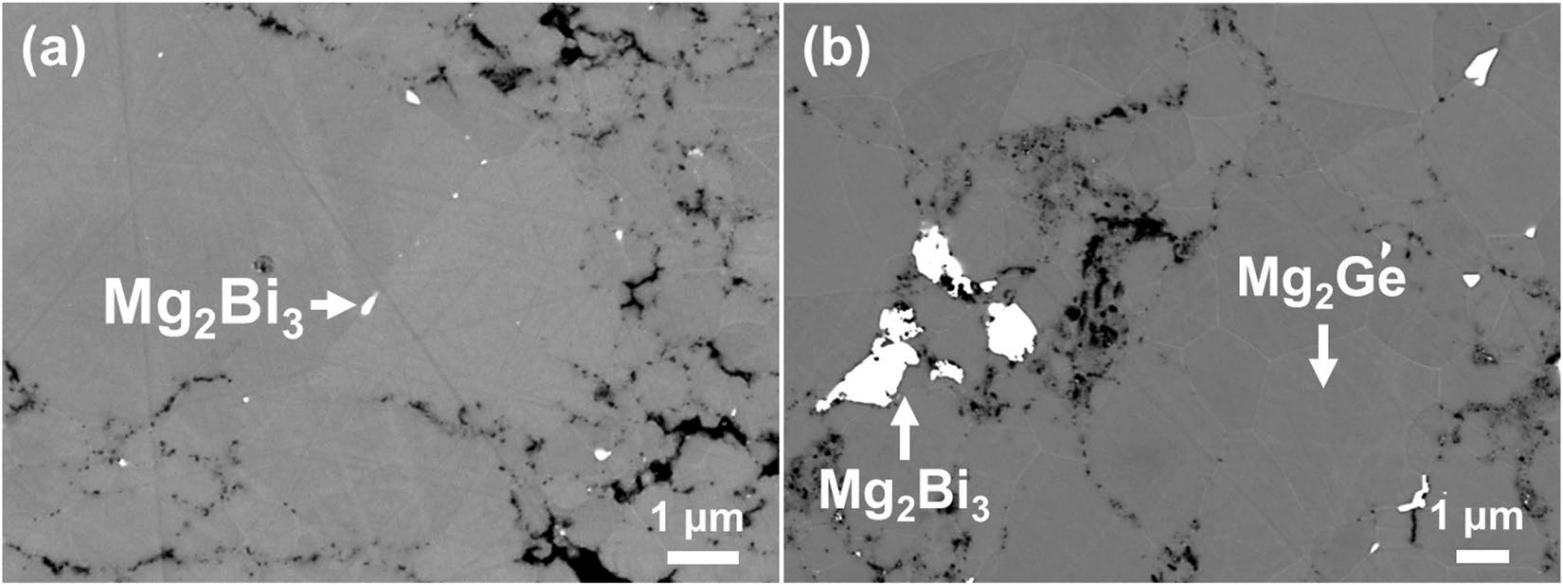
Backscattered electron micrographs of Mg_2_Ge_1−*x*_Bi_*x*_ samples for (**a**) *x* = 0.005, (**b**) *x* = 0.020. The interaction volume between the beam and the sample is taken into account, leading to the use of low acceleration voltages in order to distinguish the elements in the precipitates from the surrounding areas.

Electron microscopy of the samples revealed the presence of high atomic number precipitates, mostly at the grain boundaries in all doped samples, as shown for the *x* = 0.005 and *x* = 0.020 samples in Fig. 3. Energy dispersive spectroscopy (EDS) analysis of the precipitates and the matrix confirms that the matrix is composed of Mg and Ge, and that the precipitates are composed of Bi and Mg. No Ge was detected in the precipitates, indicating that the observed precipitates were formed by the reaction of Bi and Mg. The Mg_3_Bi_2_ phase can be detected in the XRD pattern of the doped sample with *x* = 0.020 where the concentration of this secondary phase was high enough to overcome the detection limit of the XRD technique. Although this phase was undetected by XRD analysis in sample *x* = 0.030, one can assume a larger number of Mg_2_Bi_3_ precipitates at higher concentration of added Bi. The larger number of precipitates in this sample results in higher electron scattering at interfaces and defects, explaining its lower carrier mobility^27^ (Table 1). These results suggest very limited solubility of Bi in Mg_2_Ge (maximum 0.17 at.%), below the concentration required to contribute significantly towards the charge carrier concentration.

**Figure 4:**
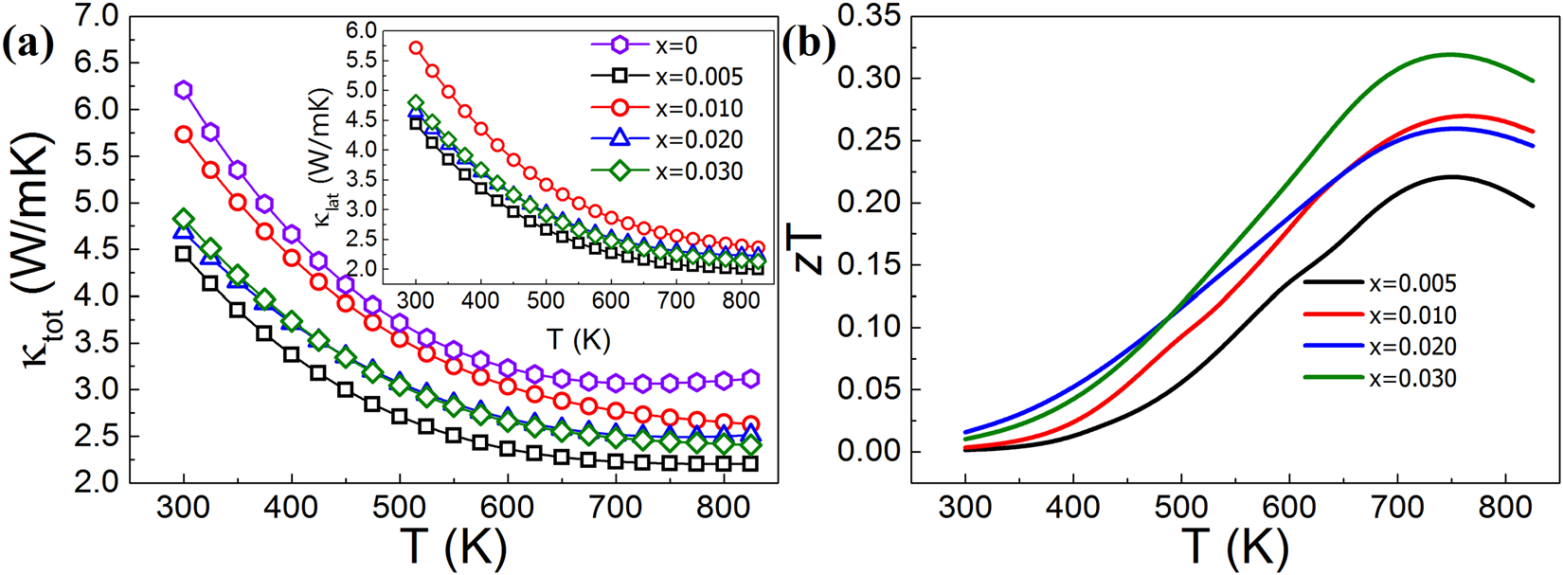
The temperature dependence of (**a**) the total thermal conductivity (inset: the lattice thermal conductivity) and (**b**) the figure-of-merit, *z*T, of the Mg_2_Ge_1−*x*_Bi_*x*_ (*x* = 0.005, 0.010, 0.020, 0.030) samples. Sample *x* = 0.005 exhibited a relative mass density of 86%, much lower than the 97% of all other samples, leading to a reduced *κ*_tot_.

The total thermal conductivity (*κ*_tot_) of the Mg_2_Ge_1−x_Bi_x_ samples decreases continuously with temperature with the exception of the intrinsic (*x* = 0) sample, which shows a slight upturn at ~700 K due to the bipolar effect; this effect is not visible in the Bi-doped samples (Fig. 4). Increased concentration of Bi dopant led to a decrease in the *κ*_tot_ of all the samples. The sample with *x* = 0.005 shows reduced thermal conductivity, which can be explained by its relative density being much lower (86%) than those of the other samples (97%). The increased porosity in this sample results in lower thermal conductivity.

The lattice thermal conductivity (*κ*_lat_) of samples is calculated by subtracting the electronic thermal conductivity (*κ*_e_) from *κ*_tot_ using the Wiedemann-Franz relation, ${\kappa }_{e}={L}_{0}T/\rho$ (where *L* is the Lorenz number and *ρ* the electrical resistivity). The Lorenz number used is the theoretical limit for degenerate semiconductors of $2.45\times {10}^{-8}$ V^2^K^−2^ ^28^, as the approximation for non-degenerate semiconductors, $L=1.5+\exp [-\frac{|S|}{116}]$ (where *L* is in 10^−8^ WΩK^−2^ and *S* in µV/K) has a negligible impact in the calculation of *κ*_lat_ for lightly doped samples^29^, calculated to be below 4% at maximum temperature for the current study samples. No significant difference between the total thermal conductivity and its lattice component was observed in the Bi-doped Mg_2_Ge samples, due to the very low charge carrier concentration (Table 1), resulting in a low electronic thermal conductivity (Fig. 4(a)). This explains the suppressed influence of the bipolar effect on the *κ*_tot_ of these samples, while it is clearly observed in the Seebeck coefficient and electrical conductivity of all the samples, as shown in Fig. 3. All doped samples show a decrease in the total thermal conductivity in relation to the intrinsic one, which suggests that Bi increases the phonon scattering by mass-difference point defect impurity scattering up to the solubility limit of Bi in Mg_2_Ge. More notably, Bi addition also contributes to the reduction of thermal conductivity through phonon scattering by precipitates, at concentrations higher than *x* = 0.005. The maximum *z*T of 0.32 was obtained for the sample with *x* = 0.030 at 750 K. The improvement in efficiency for the samples with a higher Bi content, *x* = 0.020 and 0.030, was obtained mostly due to the achievement of lower thermal conductivity (Fig. 4(b)). This results in maximum *z*T value of ~0.3, higher than the maximum reported figure of merit of ~0.2 for Sb-doped Mg_2_Ge^12^.

Bismuth, on par with antimony, is a common *n*-type dopant of group IV elements, contributing to the charge carrier density with one electron per substitution^30, 31^. Understanding the effects of Bi-doping on promising thermoelectric Mg_2_X (X = Si, Ge, Sn) compounds is therefore of great importance and interest. Excess Bi was observed to segregate at the grain boundaries of Mg_2_Si^9^ at concentrations above 0.7 at.%, considerably higher than the concentration of 0.17 at.% determined in the current study for Mg_2_Ge. This result conflicts with a theoretical study^32^ using first-principles density functional theory (DFT) calculations, which suggested both Bi and Sb as dopants for Mg_2_Ge with good solubility, due to the negative formation energies of these elements in the Ge-site substitution. The formation energy of Sb and Bi on the Ge-sites in Mg_2_Ge was found to be considerably lower than on the Mg-sites by DFT calculations. Therefore, it was suggested that these elements would substitute Ge atoms in the Mg_2_Ge structure, resulting in one free electron per substitution. The substitution of Ge by Sb should then lead to an increase in charge carrier concentration, decreasing the electrical resistivity, and also decreasing the lattice thermal conductivity due to phonon scattering on point defects^17^. These characteristics were confirmed experimentally for *n*-type Sb-doped Mg_2_Ge synthesized by a melting technique^12^ and solid-state synthesis^17^. There are no reports of Sb-rich secondary phases detected in Sb-doped Mg_2_Ge. The only stated drawback for Sb-doped Mg_2_X (X = Si, Ge, Sn) is its influence on the formation of Mg vacancies at higher concentrations, which act as holes^12, 17, 33^.

**Figure 5:**
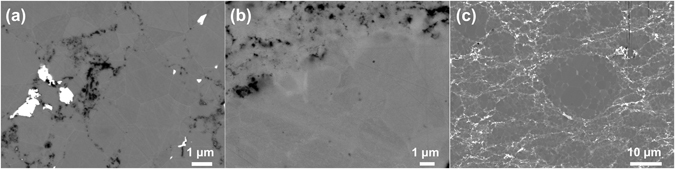
SEM/BSE micrographs of Mg_2_Ge_0.98_X_0.02_ samples: (**a**) X = Bi, (**b**) X = Sb, and (**c**) X = Bi and ion-milled.

In order to understand the differences between the Bi-doped samples of the current study and previous reports of Sb-doped Mg_2_Ge^12, 17^, a Sb-doped sample was fabricated using the one-step synthesis. The electronic transport properties of this sample have been compared with those of the Bi-doped samples and previous reports^12, 17^. Both Sb and Bi are expected to contribute with one electron per Ge atom substitution, and thus, both samples were doped with the same concentration of 0.67 at.% dopant (Mg_2_Ge_0.98_X_0.02_, X = Bi, Sb). The structure and morphology of these samples are compared in Fig. 5 by backscattered electron microscopy (BSE) SEM. The Sb-doped sample shows no signs of Sb-rich precipitates. Both samples show high porosity at the grain boundaries, which is believed to originate from the presence of intergrain MgO. These oxide particles are removed during the mechanical polishing of samples for SEM characterization. Therefore, a Bi-doped sample was prepared for SEM analysis by ion-milling to eliminate the effect of mechanical polishing. The SEM-EDS analysis (Fig. 5(c) detects intergrain Bi-rich phase, however.

**Figure 6:**
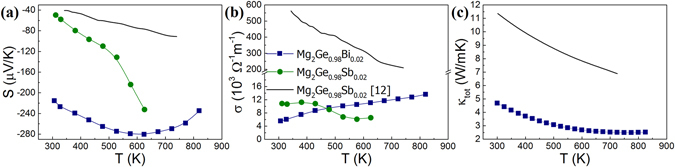
Temperature dependence of (**a**) the Seebeck coefficient, (**b**) the electrical conductivity, and (**c**) the total thermal conductivity of Mg_2_Ge_0.98_X_0.02_ (X = Bi, Sb) samples and Mg_2_Ge_0.98_Sb_0.02_^12^.

Figure 6 compares the thermoelectric properties of Bi- and Sb-doped Mg_2_Ge samples in this study with the previously reported Sb-doped Mg_2_Ge synthesized by a melting technique^12^. The Bi-doped sample exhibits a roughly constant Seebeck coefficient throughout the temperature range (Fig. 6(a)), typical behavior of an non-degenerate semiconductor, whereas the absolute value of the Seebeck coefficient of the Sb-doped sample increases with temperature, similar to the previous study^12^. The electrical conductivity of the Bi-doped sample increases with temperature, which is contrary to the negative trend for the Sb-doped one and typical behavior for a highly degenerate semiconductor (Fig. 6(b)). Both samples exhibit similar room temperature electrical conductivities. The differences might have originated from the different synthesis methods, which may result in various intrinsic point defects. Due to the volatility of Mg, the various synthesis methods require different amounts of extra Mg to compensate for its loss during heating processes. This variation in stoichiometry plays a significant role in the charge carrier concentration, to a point where it supersedes the influence of the dopant^34^ and consequently affects the transport properties of compounds. Nevertheless, these results indicate that Mg_2_Ge can be successfully doped with Sb, while Bi mostly forms precipitates with Mg, despite being theoretically postulated as a dopant equivalent to Sb. The increased phonon scattering effect introduced by the Bi-rich precipitates leads to a significantly lower thermal conductivity, as evidenced in Fig. 6(c).

Table 2 compares the room temperature characteristics of *n*-type Mg_2_Ge samples reported in the literature^12, 17^ with the Bi and Sb-doped samples of this study. Even though all the samples are similarly doped, their thermoelectric properties vary by several orders of magnitude. This is even more significant between previous reports^12, 17^, even though the synthesis method is similar for both reports. The difference is attributed to the final Mg stoichiometry, which has a significant impact, mainly on the charge carrier concentration, which arises from the different amounts of excess Mg added during synthesis^12^.

**Table 2:** Room temperature thermoelectric characteristics of Mg_2_Ge_0.98_X_0.02_ (X = Bi, Sb) samples in this study and reported in literature^12, 17^.

	Bi-doped	Sb-doped	Sb-doped	Sb-doped
σ (10^3^ Ω^−1^m^−1^)	5.5	10.9	1.2	563.2
S (µV/K)	−215.6	−49.4	−309.7	−40.6

## Conclusions

Synthesis of *n*-type Bi-doped Mg_2_Ge thermoelectric materials was performed for the first time by a one-step spark plasma sintering technique using MgH_2_ and elemental Ge. We have shown that there is very limited solubility of Bi in Mg_2_Ge, below 0.17 at.%, which results in the formation of a Mg_2_Bi_3_ secondary phase in the form of precipitates located mainly at the grain boundaries. This results in negligible improvement in the power factor of samples with added Bi due to a low doping efficiency by Bi in Mg_2_Ge and suppressed influence in the power factor due to a significantly lower electrical conductivity, despite the improved Seebeck coefficient, when compared with a report on Sb-doped Mg_2_Ge. The precipitates, however, enhanced the phonon-scattering and consequently led to a very significant reduction of the *κ*_lat_ and *κ*_tot_, achieving a *z*T of 0.32 at 750 K, higher than previously reported maximum value of 0.2 for Sb-doped Mg_2_Ge. The same synthesis method produced precipitate-free Sb-doped Mg_2_Ge alloys.
